# Pediatric Anal Pruritus and Misdiagnosis: *Dipylidium caninum* in a Spanish Child with a Review of Reported Human Cases

**DOI:** 10.4269/ajtmh.26-0208

**Published:** 2026-06-25

**Authors:** Paola Cociancic, Verónica Prieto, Jacklyn Comas, J. Guillermo Esteban

**Affiliations:** ^1^ Área de Parasitología, Departamento de Farmacia y Tecnología Farmacéutica y Parasitología, Facultad de Farmacia y Ciencias de la Alimentación, Universidad de Valencia, Valencia, Spain;; ^2^ Salud y Comunidad, Ciencias de la Salud, Ciencia, Tecnología e Innovación en Salud, Universidad Tecnológica del Choco Diego Luis Córdoba, Quibdó, Colombia

## Abstract

Intestinal helminth infections in children remain a significant public health problem worldwide. Among these, those caused by cestodes can be misdiagnosed, as occurs with dipylidiasis, a zoonosis caused by *Dipylidium caninum*. Although common in dogs and cats, human infection is rare and may be misdiagnosed as *Enterobius vermicularis* due to overlapping symptoms. We present the case of a 3-year-old Spanish boy with intense anal itching, treated twice with mebendazole for presumed pinworm infection without improvement. Persistent symptoms, anorexia, and weight loss prompted a parasitological examination. Microscopic examination of whitish structures in the feces revealed gravid proglottids with bilateral genital pores and egg packets containing hexacanth embryos, confirming *D. caninum* infection. Epidemiological investigation suggested exposure to a relative’s dog as the probable source. Treatment with paromomycin sulfate resulted in complete cure. This case highlights the importance of recognizing the characteristic morphology of *D. caninum* to avoid misdiagnosis and inappropriate therapy.

## INTRODUCTION

Intestinal parasitic infections remain a major public health problem worldwide, particularly in tropical regions. The WHO estimates that more than 1.5 billion people are at risk of contracting helminthiases, primarily soil-transmitted infections.^1^ In addition to these infections, other helminths (including trematodes and cestodes) are less common in humans and are transmitted through intermediate hosts, as in some cestodes.

The family Dilepidiidae (Cestoda, Cyclophyllidea) includes species responsible for the zoonotic parasitic disease dipylidiasis, with *Dipylidium caninum* as the main etiological agent. This cestode requires an arthropod intermediate host—such as fleas or lice—for transmission, with humans acquiring infection accidentally through ingestion of parasitized hosts carrying larval stages. Domestic and wild canids and felines serve as definitive hosts, whereas human infections occur mainly in infants and young children because of their close contact with pets.^2^ Diagnosis can be challenging, particularly for pathologists and clinicians with limited experience, and *D. caninum* infections are often misdiagnosed as *Enterobius vermicularis* (pinworm) infection due to similar symptoms such as anal pruritus.^3^

We report the case of a preschool-age Spanish child who was initially treated twice for presumed pinworm infection before *D. caninum* was definitively confirmed. Based on this case, previously published reports of accidental human dipylidiasis were compiled to examine patterns of misdiagnosis, diagnostic challenges, and therapeutic management.

## CASE PRESENTATION

In October 2024, a 3-year-old Spanish boy presented to a pediatrician with intense anal pruritus. The child was treated with mebendazole syrup (100 mg/5 mL), with a second dose administered after 2 weeks. In November, symptoms persisted, accompanied by anorexia and weight loss. The pediatrician attributed this to presumed reinfection at kindergarten.

**Figure 1.**
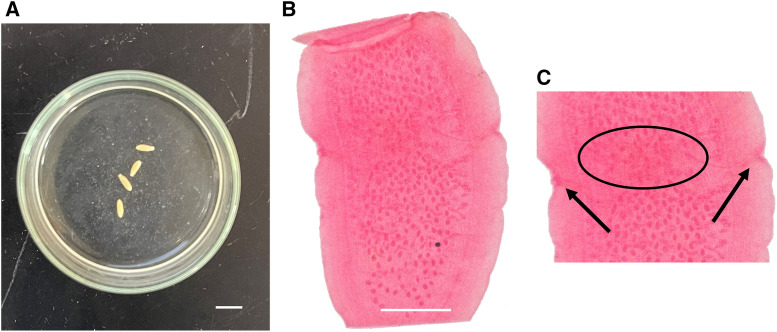
*Dipylidium caninum* from a pediatric patient. (**A**) Proglottids expelled by the patient (scale bar = 1 cm). (**B**) Carmine-stained gravid proglottid mounted in Canada balsam (scale bar = 1 mm). (**C**) Detail of the compartmentalized uterus (circle) and paired lateral genital pores (arrows).

**Figure 2.**
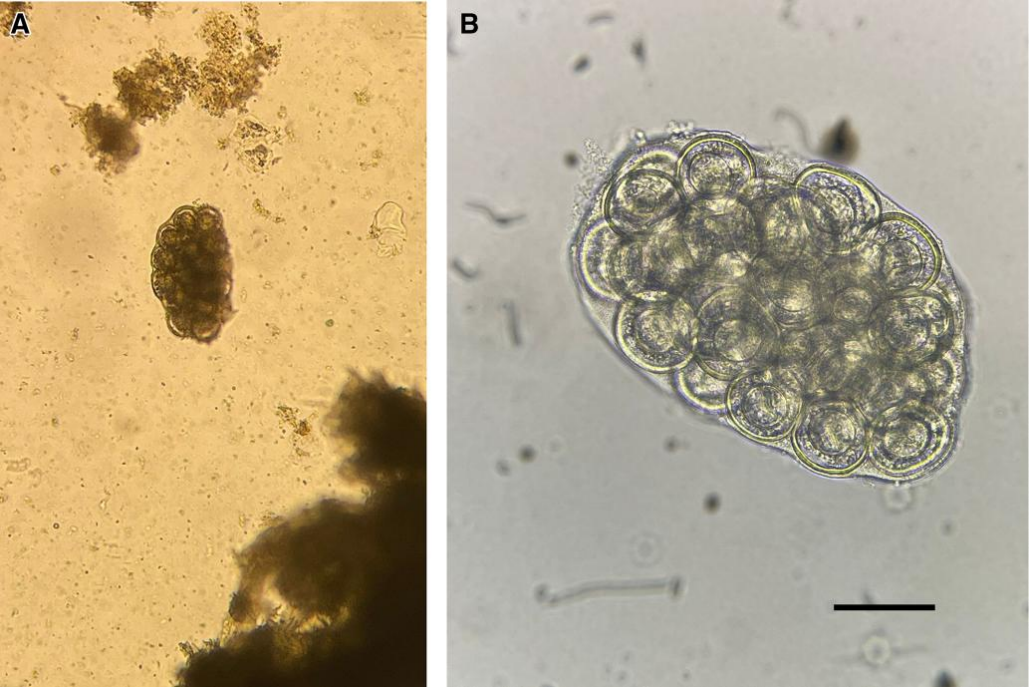
Eggs packets of *Dipylidium caninum* in fecal smears. (**A**) Packets containing 22 eggs at low power. (**B**) Packet containing 24 eggs mounted a drop of Amann’s Lactophenol. Each egg contains an oncosphere (scale bar = 50 *µ*m).

Within a week, the child’s mother observed small white structures in the stool (Figure 1A), initially thought to be food remnants. She consulted a pharmacist, who referred the case to the Department of Parasitology, Faculty of Pharmacy and Food Sciences, University of Valencia. Three consecutive fecal samples and three Graham tape tests, along with the recovered structures, were analyzed. Microscopic examination of carmine-stained proglottids mounted in Canada balsam revealed gravid proglottids with two lateral genital pores and a compartmentalized uterus containing egg clusters (Figure 1B and C). Graham tape tests for pinworm detection were negative. Formaldehyde–ethyl acetate–concentrated fecal samples showed two egg packets containing clusters of 22 to 24 spherical eggs (25–40 *µ*m in diameter) with hexacanth embryos bearing six delicate hooklets (Figure 2A and B). The findings confirmed *D. caninum* infection.

Neither the parents nor the child’s sibling were infected, and the family had no household pets. Treatment with paromomycin sulfate (Humatín® [Pfizer], Waylis Therapeutics LLC, Rahway, NJ, USA) was administered orally at 50 mg/kg/day divided into three doses, and follow-up stool examinations at 7, 15, 30, and 60 days were negative, confirming parasitological cure.

## DISCUSSION

*Dipylidium caninum* is a cyclophyllidean tapeworm measuring 10 to 70 cm in length. The scolex is conical, with four suckers and a retractile rostellum armed with several rings of small spines. Gravid proglottids are longer than wide (23 × 8 mm) and feature two genital pores, one on each lateral margin, a key feature distinguishing them from *Inermicapsifer madagascariensis*, which has a single genital pore in the middle of each lateral margin. Both species have gravid proglottids divided into multiple compartments, each containing eggs with six-hooked embryos—10 to 30 eggs per packet in *Dipylidium* versus 8 to 15 in *Inermicapsifer*.^2^ In addition, *Dipylidium* has a characteristic tapered shape resembling seeds or rice grains.

This cestode requires two hosts to complete its life cycle. Domestic and feral dogs, other canids, and cats are definitive hosts, whereas humans may occasionally serve as accidental definitive hosts. Fleas (*Ctenocephalides* spp., *Pulex irritans*) and lice (*Trichodectes canis*, *Felicola subrostratus*) serve as intermediate hosts.^3^ Gravid proglottids are expelled individually or in short chains, or they may emerge spontaneously from the anus. After ingestion by intermediate hosts, six-hooked embryos develop into procercoid larvae and subsequently into cysticercoid metacestodes in the hemocoel, where they remain until the adult flea or louse matures. Infection is acquired by ingestion of infected intermediate hosts, with maturation into adult tapeworms occurring within 3 to 4 weeks.

Although *D. caninum* infection is rare in humans, it is the most common cestode in dogs in Spain.^4^^,^^5^ Published human cases compiled in Table 1 show that accidental infection has been reported in Africa, America, Asia, and Europe over the past 25 years,^6^^–^^35^ with children being the most frequently affected, likely because of close contact with animals and inadequate hand hygiene. Infection may also occur if fleas or lice contaminate food. Published cases often report infected pets harboring fleas.^9^^,^^15^^,^^21^^,^^32^ In our case, the child had no household pets, and the likely source was a cousin’s dog diagnosed with dipylidiasis. Similarly, several reported cases describe infections in children without pets at home, suggesting exposure through contact with parasitized animals in relatives’ homes or other environments.

**Table 1:** Reported human cases of *Dipylidium caninum* infection worldwide over the last 25 years

Country	Patient Age	Clinical History	Hematological/ Biochemical Findings	Previous Misdiagnoses/ Treatment	Diagnosis	Treatment	Animal Exposure and/or Flea Infestation	Reference
Africa								
Egypt	1 preschooler and 3 schoolers	Gastrointestinal discomfort, anal pruritus (*n* = 2)	ND	ND	Stool proglottids, direct smear and formol-ether concentration	ND	Pets at home, risk factor; fleas ND	6
Ethiopia	3 years	Nausea, vomiting, diarrhea	ND	ND	Proglottids and eggs by flotation technique (MgSO_4_ 1.2)	ND	ND	7
Ghana	27 years	1st trimester pregnant. Intermittent vomiting, slight headache, bloated stomach	Eosinophilia	No	Eggs by direct smear	Praziquantel 1 dose, 600 mg	No pets	8
America								
Chile	2 years	Proglottids in stool, abdominal pain	ND	Initial stool exam negative; *Hymenolepis diminuta* previously diagnosed	Proglottids and eggs packets	Praziquantel 1 dose	Dogs and cat; fleas present	9
	6 years	Proglottids in stool	ND	No	Proglottids and eggs packets	ND	ND	10
Colombia	11 months	Proglottids in stool	No	Pinworm? Treated with pyrantel, albendazole, metronidazole	Proglottids and eggs packets by direct smear; molecular analysis	Praziquantel 3 doses, 25 mg/kg/day × 2 days	Dog, parasitized and dewormed; fleas present	11
	40 years	Proglottids in anus	ND	Worm infection treated with albendazole	Proglottids	Praziquantel 1 dose, 600 mg	No pets	12
Cuba	1 year	Proglottids in stool, diarrhea, insomnia, nighttime pruritus, skin lesions, reduced appetite, weight loss	ND	No	Proglottids and eggs packets	ND	ND	13
Mexico	18 months	Proglottids in stool	No	Worm infection treated with albendazole	Proglottids and eggs packets	Praziquantel 25 mg/kg, mild laxative 1 hour later, soft diet 2 days	No pets; contact with family dog; fleas ND	14
USA	3 months	Proglottids in nappy, increased feedings, irritability, nappy rash	ND	Initial stool exam negative; pinworm?	Proglottids and eggs packets	Praziquantel 2 doses, 10 mg/kg, 2-week interval	Dogs, dewormed; fleas present	15
	2 years	Proglottids in stool, perianal pruritus	ND	Six stool samples negative; pinworm treated with albendazole	History and visual footage	Praziquantel 1 dose, 10 mg/kg	Dogs and cat; no fleas	16
	6 months	Proglottids in stool	ND	Vegetable seeds	Proglottids and eggs packets	ND	No pets; contact with daycare dog; fleas ND	17
	2 years	Proglottids in stool, perianal pruritus	ND	Initial stool exam negative	Proglottids and eggs packets	Praziquantel 1 dose	Cats, parasitized and dewormed; fleas ND	18
	2 years	Proglottids in perianal area, mild epigastric pain	ND	Pinworm treated with mebendazole	Proglottids and eggs packets	Praziquantel 1 dose, 150 mg	Cats, parasitized and dewormed; fleas ND	19
	4 months	Proglottids in nappy, severe irritability, apparent abdominal pain, agitation	Slight eosinophilia	Initial stool exam negative; pinworm treated with mebendazole	Proglottids and eggs packets	Praziquantel 1 dose	Dog, parasitized and dewormed; fleas ND	20
Venezuela	11 months	Proglottids in anus, reduced appetite, abdominal pain, low weight/height	Light hypoalbuminemia, decrease sodium and calcium, high IgE, slight eosinophilia, anemia	Not previously diagnosed by other laboratories	Proglottids and eggs packets by wet mount and formol-ether concentration	Praziquantel 1 dose, 10 mg/kg	Dog, parasitized and dewormed; fleas present	21
Asia								
China	17 months	Proglottids in stool, mild diarrhea	Anemia, high serum IgE	Pinworm treated with albendazole	Proglottids in stool	Praziquantel 1 dose, 25 mg/kg, 1 hour later Epsom salts	Dog; fleas suspected	22
India	10 years	Pruritic red lesions, colicky abdominal pain, fullness of the epigastrium, occasional nausea and vomiting, low weight	ND	No	Proglottids and egg packets by wet mount	Praziquantel pamoate 1 dose, 10 mg/kg	Habit of handling cats and street dogs	23
	50 years	Proglottids in stool, lower abdominal pain, dry cough, occasional vomiting, stools occasionally loose	Low hematocrit and platelet, high erythrocyte sedimentation rate	Treated with albendazole	Proglottids and egg packets by wet mount	Praziquantel 600 mg daily × 5 days	Feeding stray dogs	24
	4 years	Proglottids in stool, anal pruritus, recurrent abdominal pain	Low hematocrit	Pinworm treated with albendazole and antihistamines	Proglottids and egg packets by wet mount	Praziquantel pamoate 1 dose, 10 mg/kg	Cats; handling street dogs; fleas ND	25
	9 years	Nausea, vomiting, diarrhea, persistent low-grade fever	Low hematocrit, mean corpuscular volume, mean corpuscular hemoglobin	No	Proglottids and egg packets by wet mount	Praziquantel	Fleas suspected	26
Japan	17 months	Proglottids in stool, abdominal pain, diarrhea, night dysphoria	ND	Pinworm treated with pyrantel pamoate	Proglottids and eggs packets	Praziquantel 1 dose	Dog and cat, parasitized and dewormed; fleas present	27
	3 years	Proglottids in nappy	ND	Coprological exam negative	Visual footage	None	Cat, parasitized and dewormed; fleas ND	28
Europe								
Greece	7 months and parents	Proglottids in stool	No	No	Proglottids	Praziquantel 100 mg × 3/day, 15 days later praziquantel + niclosamide 500 mg	Stray cat contact	29
	10 children (7 months– 10 years)	Proglottids in stool (*n* = 3), distress during defecation (*n* = 1), poor weight gain, chronic abdominal pain (*n* = 2), chronic diarrhea (*n* = 4)	Leukocytosis severe eosinophilia (*n* = 1)	Probable prior pinworm infection	Proglottids and eggs	Praziquantel 1 dose, 20 mg/kg (*n* = 7), praziquantel 2 doses, 2-week interval, 20 mg/kg (*n* = 1), praziquantel 2 doses, 1-month interval, 20 mg/kg (*n* = 1), praziquantel 2 doses, 2-week interval, 20 mg/kg (*n* = 1), niclosamide 1 dose, 500 mg, 2 months after praziquantel (*n* = 2)	Dogs (*n* = 4), stray cat contact (*n* = 1); fleas ND	30
Poland	2 years	Proglottids on underwear, in water while bathing and in stool, abdominal pain, sleep disorders, reduced appetite, hyperactivity, occasional slimy stool	No	Worm treated with pyrantel and albendazole	Proglottids and eggs packets by direct preparation, Kato thick smear, and decantation	Praziquantel 1 dose, 10 mg/kg	Dogs and cats, parasitized and dewormed; stray cat contact; fleas present	31
Russia	8 children (11 months– 12 years) and 27 years adult	Proglottids in stool	ND	Parents treated independently for worm/pinworm infection with levamisole, mebendazole, albendazole, and pyrantel	Proglottids	Praziquantel 1 dose, 15 mg/kg	Dogs and cats, parasitized and dewormed; fleas present	32
Spain	11 months	Diarrhea, fever, green and liquid feces (5 times/day), sadness, irritability, abdominal pain, enlarged abdomen	ND	No	Egg packets	Praziquantel 1 dose, 150 mg	Dogs; fleas ND	33
	9 months	Proglottids in stool	ND	No	Proglottids and egg packets	Praziquantel 1 dose, 10 mg/kg	Dog, parasitized; fleas suspected	34
Turkey	26 years	Kidney transplant patient. Prolonged diarrhea, abdominal pain	No	Antimicrobial treatment	Eggs	Niclosamide 500 mg × 4/day for 1 day	No pets	35

ND = not described.

Human infection is often asymptomatic; however, infants may present with nocturnal irritability, anorexia, weight loss, or failure to thrive. Symptoms often go unnoticed until parents observe motile structures in the feces.^9^^,^^11^^,^^15^^,^^28^ Clinical manifestations may include abdominal pain, colic, episodic vomiting, diarrhea, anal pruritus, or rash.^13^^,^^23^^–^^25^^,^^27^ Less frequently, urticaria or eosinophilia may occur.^8^^,^^20^ Symptoms are often underestimated, and severe cases have been described, such as a kidney transplant patient treated with antimicrobial therapy for diarrhea and abdominal pain.^35^ Reports in the literature also describe occasional hematological alterations, including eosinophilia,^8^^,^^20^^,^^21^^,^^30^ hypoalbuminemia,^21^ elevated IgE levels,^21^^,^^22^ or anemia,^21^^,^^22^^,^^24^^–^^26^ although such findings are inconsistent and not always documented.

Although uncommon, *D. caninum* infections may be first detected by pathologists or laboratory personnel in clinical microbiology or surgical pathology laboratories. Familiarity with the diagnostic morphology—gravid double-pored proglottids or egg packets with six-hooked embryos—is crucial. Human diagnosis is challenging, frequently confused with pinworm infection, which may lead to inappropriate treatment (e.g., albendazole, mebendazole, pyrantel pamoate) and prolonged infection.^11^^,^^14^^,^^16^^,^^19^^,^^20^^,^^22^^,^^25^^,^^27^^,^^32^ Our compilation of published cases shows that misdiagnosis is frequent, with many patients initially treated for presumed *E. vermicularis* infection^11^^,^^16^^,^^19^^,^^20^^,^^22^^,^^25^^,^^27^^,^^32^ or other intestinal parasitosis such as *Hymenolepis* infection.^9^ In several reports, patients received multiple ineffective anthelmintic or antimicrobial treatments before the correct diagnosis was established. In addition, proglottids occasionally have been mistaken for food particles or seeds.^17^ Distinguishing parasitic structures from pseudoparasites or fecal artifacts is a common challenge, especially for less experienced personnel.^36^^,^^37^ Serological methods, mainly ELISA, can detect antibodies indicating current or past infection, although cross-reactions may occur.^38^ Molecular methods have recently improved diagnostic accuracy.^11^

Praziquantel (Biltricide® 5–10 mg/kg, single dose) is the treatment of choice for humans.^39^ It disintegrates the tapeworm in the intestine, preventing its observation in feces, and is generally well tolerated. However, its use in pregnant or breastfeeding women is not recommended because it is excreted in breast milk.^34^ Niclosamide (Yomesan® 2 g, single dose in adults, 0.05–2 g/kg in children) is an alternative but requires intestinal preparation with a liquid diet.^39^ In Spain, these drugs are not routinely available, so paromomycin sulfate is commonly used.^40^^,^^41^ In the cases compiled in Table 1, praziquantel was the most frequently used treatment and was effective in resolving infection in nearly all patients, occasionally combined with niclosamide or other supportive measures.

Given the worldwide distribution of this cestode, a One Health approach is essential, emphasizing health education for children and animal owners as well as control of intermediate hosts in pets and the surrounding environment. Awareness among medical and veterinary professionals regarding the diagnostic and therapeutic challenges of dipylidiasis should therefore be increased.
